# Supplementary material for: Possible Involvement of Long Non-Coding RNAs GNAS-AS1 and MIR205HG in the Modulation of 5-Fluorouracil Chemosensitivity in Colon Cancer Cells through Increased Extracellular Release of Exosomes
Source: Noncoding RNA. 2024 Apr 15;10(2):25. doi: 10.3390/ncrna10020025 (PMC11054952; doi:10.3390/ncrna10020025)
Supplement: Supplementary file 1 [file ncrna-10-00025-s001.zip › ncrna-2535327-supplementary.pdf]

TABLE S1

List of Upregulated Genes in the cDNA Microarray Dataset

Gene Symbol	Description	Fold-change	*p*-value
**GNAS-AS1**	Homo sapiens GNAS antisense RNA 1 (GNAS-AS1), antisense RNA [NR_002785]	56.87	0.0456
**FBP1**	Homo sapiens fructose-1,6-bisphosphatase 1 (FBP1), transcript variant 1, mRNA [NM_000507]	51.82	0.0491
**MIR205HG**	Homo sapiens MIR205 host gene (non-protein coding) (MIR205HG), mRNA [NM_001104548]	39.70	0.0491
**LOC102723721**	PREDICTED: Homo sapiens uncharacterized LOC102723721 (LOC102723721), ncRNA [XR_424100]	29.62	0.0477
**SMOC2**	Homo sapiens SPARC related modular calcium binding 2 (SMOC2), transcript variant 1, mRNA [NM_022138]	20.07	0.0456
**CCDC106**	Homo sapiens coiled-coil domain containing 106 (CCDC106), mRNA [NM_013301]	18.62	0.0456
**ARHGEF26**	Homo sapiens Rho guanine nucleotide exchange factor (GEF) 26 (ARHGEF26), transcript variant 1, mRNA [NM_001251962]	18.51	0.0456
**KCTD12**	Homo sapiens potassium channel tetramerization domain containing 12 (KCTD12), mRNA [NM_138444]	18.04	0.0491
**C11orf39**	Homo sapiens chromosome 11 open reading frame 39 (C11orf39), long non-coding RNA [NR_126159]	17.18	0.0456
**GAGE7**	Homo sapiens G antigen 7 (GAGE7), mRNA [NM_021123]	16.43	0.0491
**PER3**	Homo sapiens period circadian clock 3 (PER3), transcript variant 4, mRNA [NM_016831]	13.86	0.0456
**LINC00668**	Homo sapiens long intergenic non-protein coding RNA 668 (LINC00668), long non-coding RNA [NR_034100]	13.28	0.0456
**ZNF44**	zinc finger protein 44 [Source:HGNC Symbol;Acc:HGNC:13110] [ENST00000483826]	12.53	0.0456
**CCDC149**	Homo sapiens coiled-coil domain containing 149 (CCDC149), transcript variant 1, mRNA [NM_173463]	10.37	0.0491
**MIR205HG**	Homo sapiens MIR205 host gene (non-protein coding) (MIR205HG), mRNA [NM_001104548]	9.40	0.0456
**MPP1**	Homo sapiens membrane protein, palmitoylated 1, 55kDa (MPP1), transcript variant 1, mRNA [NM_002436]	9.35	0.0485
**PRG2**	Homo sapiens proteoglycan 2, bone marrow (natural killer cell activator, eosinophil granule major basic protein) (PRG2), transcript variant 1, mRNA [NM_002728]	9.04	0.0456
**CSF2**	Homo sapiens colony stimulating factor 2 (granulocyte-macrophage) (CSF2), mRNA [NM_000758]	8.35	0.0456
**RASSF2**	Homo sapiens Ras association (RalGDS/AF-6) domain family member 2 (RASSF2), transcript variant 1, mRNA [NM_014737]	7.28	0.0456

**TCAM1P**	Homo sapiens testicular cell adhesion molecule 1, pseudogene (TCAM1P), non-coding RNA [NR_002947]	7.10	0.0491
**RDX**	Homo sapiens radixin (RDX), transcript variant 1, mRNA [NM_001260492]	6.96	0.0491
**RTKN2**	Homo sapiens cDNA FLJ39352 fis, clone PEBLM2001312, weakly similar to Homo sapiens rhotekin mRNA. [AK096671]	6.40	0.0456
**lnc-FARS2-2**	Homo sapiens cDNA clone IMAGE:4523522, partial cds. [BC036859]	6.32	0.0456
**NOS3**	Homo sapiens nitric oxide synthase 3 (endothelial cell) (NOS3), transcript variant 2, mRNA [NM_001160109]	6.26	0.0456
**TPM3**	Homo sapiens tropomyosin 3 (TPM3), transcript variant Tpm3.7, mRNA [NM_001043352]	6.03	0.0491
**MROH6**	Homo sapiens maestro heat-like repeat family member 6 (MROH6), mRNA [NM_001100878]	5.75	0.0491
**CRIP1**	Homo sapiens cysteine-rich protein 1 (intestinal) (CRIP1), mRNA [NM_001311]	5.75	0.0491
**GUSBP1**	Homo sapiens glucuronidase, beta pseudogene 1 (GUSBP1), transcript variant 1, non-coding RNA [NR_027026]	5.70	0.0456
**FHDC1**	Homo sapiens FH2 domain containing 1 (FHDC1), mRNA [NM_033393]	5.54	0.0491
**LOC100506379**	AF064804 transcription factor SUPT3H {Homo sapiens} (exp=-1; wgp=0; cg=0), partial (5%) [THC2635298]	5.39	0.0491
**RGS9**	Homo sapiens regulator of G-protein signaling 9 (RGS9), transcript variant 1, mRNA [NM_003835]	5.19	0.0491
**DUSP5P1**	Homo sapiens dual specificity phosphatase 5 pseudogene 1 (DUSP5P1), non-coding RNA [NR_002834]	5.17	0.0491
**HSPA1B**	Homo sapiens heat shock 70kDa protein 1B (HSPA1B), mRNA [NM_005346]	5.13	0.0456
**TMEM19**	Homo sapiens transmembrane protein 19 (TMEM19), mRNA [NM_018279]	5.02	0.0456
**LOC100507002**	Homo sapiens uncharacterized LOC100507002 (LOC100507002), long non-coding RNA [NR_110801]	4.92	0.0491
**SNX21**	Homo sapiens sorting nexin family member 21 (SNX21), transcript variant 4, mRNA [NM_001042633]	4.85	0.0456
**TUBB**	Homo sapiens tubulin, beta class I (TUBB), transcript variant 2, mRNA [NM_178014]	4.79	0.0491
**NCAPH**	Homo sapiens non-SMC condensin I complex, subunit H (NCAPH), transcript variant 1, mRNA [NM_015341]	4.77	0.0491
**ZFAT**	zinc finger and AT hook domain containing [Source:HGNC Symbol;Acc:HGNC:19899] [ENST00000522974]	4.76	0.0485
**SMC1A**	Homo sapiens structural maintenance of chromosomes 1A (SMC1A), transcript variant 1, mRNA [NM_006306]	4.69	0.0491
**TRDMT1**	Homo sapiens tRNA aspartic acid methyltransferase 1 (TRDMT1), mRNA [NM_004412]	4.56	0.0491
**TUBB**	Homo sapiens tubulin, beta class I (TUBB), transcript variant 1, mRNA [NM_001293212]	4.56	0.0491
**RCN3**	Homo sapiens reticulocalbin 3, EF-hand calcium binding domain (RCN3), mRNA [NM_020650]	4.53	0.0459
**LINC01431**	Homo sapiens long intergenic non-protein coding RNA 1431 (LINC01431), long non-coding RNA [NR_109884]	4.50	0.0491

**lnc-HORMAD2-1**	LNCipedia lincRNA (lnc-HORMAD2-1), lincRNA [lnc-HORMAD2-1:1]	4.37	0.0456
**XLOC_l2_007176**	BROAD Institute lincRNA (XLOC_l2_007176), lincRNA [TCONS_l2_00013434]	4.35	0.0491
**LOC646014**	Homo sapiens cDNA FLJ44593 fis, clone BLADE2002744. [AK126557]	4.32	0.0491
**LRRC45**	leucine rich repeat containing 45 [Source:HGNC Symbol;Acc:HGNC:28302] [ENST00000306688]	4.32	0.0491
**OBSCN**	Homo sapiens obscurin, cytoskeletal calmodulin and titin-interacting RhoGEF (OBSCN), transcript variant IC, mRNA [NM_001271223]	4.30	0.0491
**HMGCR**	Homo sapiens 3-hydroxy-3-methylglutaryl-CoA reductase (HMGCR), transcript variant 1, mRNA [NM_000859]	4.19	0.0491
**DHCR24**	Homo sapiens 24-dehydrocholesterol reductase (DHCR24), mRNA [NM_014762]	4.16	0.0456
**WDR89**	Homo sapiens WD repeat domain 89 (WDR89), transcript variant 1, mRNA [NM_001008726]	4.11	0.0492
**lnc-GPN2-1**	LNCipedia lincRNA (lnc-GPN2-1), lincRNA [lnc-GPN2-1:1]	4.07	0.0491
**FOXN1**	Homo sapiens forkhead box N1 (FOXN1), mRNA [NM_003593]	4.00	0.0478
**ARMC7**	armadillo repeat containing 7 [Source:HGNC Symbol;Acc:HGNC:26168] [ENST00000245543]	3.92	0.0491
**MICB**	Homo sapiens MHC class I polypeptide-related sequence B (MICB), transcript variant 1, mRNA [NM_005931]	3.91	0.0478
**lnc-PRL-2**	LNCipedia lincRNA (lnc-PRL-2), lincRNA [lnc-PRL-2:1]	3.86	0.0492
**CPSF6**	Homo sapiens cleavage and polyadenylation specific factor 6, 68kDa (CPSF6), transcript variant 1, mRNA [NM_007007]	3.82	0.0491
**lnc-VAPA-1**	LNCipedia lincRNA (lnc-VAPA-1), lincRNA [lnc-VAPA-1:1]	3.80	0.0491
**PSMD3**	Homo sapiens proteasome (prosome, macropain) 26S subunit, non-ATPase, 3 (PSMD3), mRNA [NM_002809]	3.77	0.0491
**EEF2K**	Homo sapiens eukaryotic elongation factor 2 kinase (EEF2K), mRNA [NM_013302]	3.77	0.0491
**ZNF444**	Homo sapiens zinc finger protein 444 (ZNF444), transcript variant 2, mRNA [NM_001253792]	3.77	0.0491
**GYS1**	Homo sapiens glycogen synthase 1 (muscle) (GYS1), transcript variant 1, mRNA [NM_002103]	3.74	0.0491
**SCP2**	Homo sapiens sterol carrier protein 2 (SCP2), transcript variant 2, mRNA [NM_001007098]	3.70	0.0491
**RETSAT**	retinol saturase (all-trans-retinol 13,14-reductase) [Source:HGNC Symbol;Acc:HGNC:25991] [ENST00000490291]	3.63	0.0491
**ADAMTS17**	ADAM metallopeptidase with thrombospondin type 1 motif, 17 [Source:HGNC Symbol;Acc:HGNC:17109] [ENST00000378898]	3.57	0.0491
**MRS2**	Homo sapiens MRS2 magnesium transporter (MRS2), transcript variant 1, mRNA [NM_001286264]	3.57	0.0491
**FTSJ3**	Homo sapiens FtsJ homolog 3 (E. coli) (FTSJ3), mRNA [NM_017647]	3.49	0.0491

**SLC25A10**	Homo sapiens solute carrier family 25 (mitochondrial carrier; dicarboxylate transporter), member 10 (SLC25A10), transcript variant 2, mRNA [NM_012140]	3.46	0.0491
**LTB4R2**	Homo sapiens leukotriene B4 receptor 2 (LTB4R2), transcript variant 1, mRNA [NM_019839]	3.46	0.0491
**SNX21**	Homo sapiens sorting nexin family member 21 (SNX21), transcript variant 4, mRNA [NM_001042633]	3.44	0.0491
**IMP3**	Homo sapiens IMP3, U3 small nucleolar ribonucleoprotein (IMP3), mRNA [NM_018285]	3.43	0.0491
**PIK3CD**	Homo sapiens phosphatidylinositol-4,5-bisphosphate 3-kinase, catalytic subunit delta (PIK3CD), mRNA [NM_005026]	3.37	0.0491
**DNAJA1**	Homo sapiens DnaJ (Hsp40) homolog, subfamily A, member 1 (DNAJA1), mRNA [NM_001539]	3.35	0.0491
**lnc-RP11-863N1.2.1-3**	LNCipedia lincRNA (lnc-RP11-863N1.2.1-3), lincRNA [lnc-RP11-863N1.2.1-3:1]	3.31	0.0491
**USP32**	ubiquitin specific peptidase 32 [Source:HGNC Symbol;Acc:HGNC:19143] [ENST00000585720]	3.29	0.0491
**LOC100287728**	Homo sapiens uncharacterized LOC100287728 (LOC100287728), long non-coding RNA [NR_103770]	3.23	0.0491
**ZNF20**	Homo sapiens zinc finger protein 20 (ZNF20), transcript variant 1, mRNA [NM_021143]	3.20	0.0491
**NOC2L**	Homo sapiens nucleolar complex associated 2 homolog (S. cerevisiae) (NOC2L), mRNA [NM_015658]	3.16	0.0491
**NDUFA3**	Homo sapiens NADH dehydrogenase (ubiquinone) 1 alpha subcomplex, 3, 9kDa (NDUFA3), mRNA [NM_004542]	3.12	0.0491
**C14orf2**	Homo sapiens chromosome 14 open reading frame 2 (C14orf2), transcript variant 2, mRNA [NM_001127393]	3.03	0.0491
**NKAPP1**	Homo sapiens NFKB activating protein pseudogene 1 (NKAPP1), non-coding RNA [NR_027131]	2.98	0.0491
**SPATA2**	Homo sapiens spermatogenesis associated 2 (SPATA2), transcript variant 1, mRNA [NM_006038]	2.96	0.0491
**XLOC_l2_008783**	BROAD Institute lincRNA (XLOC_l2_008783), lincRNA [TCONS_l2_00016499]	2.96	0.0491
**PARP10**	Homo sapiens poly (ADP-ribose) polymerase family, member 10 (PARP10), mRNA [NM_032789]	2.94	0.0491
**RECQL4**	Homo sapiens RecQ protein-like 4 (RECQL4), mRNA [NM_004260]	2.93	0.0492
**POLR2L**	Homo sapiens polymerase (RNA) II (DNA directed) polypeptide L, 7.6kDa (POLR2L), mRNA [NM_021128]	2.92	0.0491
**IGFALS**	Homo sapiens insulin-like growth factor binding protein, acid labile subunit (IGFALS), transcript variant 2, mRNA [NM_004970]	2.91	0.0491
**HOXB6**	Homo sapiens homeobox B6 (HOXB6), mRNA [NM_018952]	2.88	0.0491
**GPR180**	Homo sapiens G protein-coupled receptor 180 (GPR180), mRNA [NM_180989]	2.83	0.0491
**TFAM**	Homo sapiens transcription factor A, mitochondrial (TFAM), transcript variant 1, mRNA [NM_003201]	2.83	0.0491

**DNAAF5**	Homo sapiens dynein, axonemal, assembly factor 5 (DNAAF5), transcript variant 1, mRNA [NM_017802]	2.78	0.0491
**ZBED1**	Homo sapiens zinc finger, BED-type containing 1 (ZBED1), transcript variant 3, mRNA [NM_001171135]	2.76	0.0491
**CD2AP**	Homo sapiens CD2-associated protein (CD2AP), mRNA [NM_012120]	2.75	0.0491
**EMX2OS**	Homo sapiens EMX2 opposite strand/antisense RNA (EMX2OS), long non-coding RNA [NR_002791]	2.75	0.0491
**EVI2A**	Homo sapiens ecotropic viral integration site 2A (EVI2A), transcript variant 1, mRNA [NM_001003927]	2.74	0.0491
**CENPI**	centromere protein I [Source:HGNC Symbol;Acc:HGNC:3968] [ENST00000403304]	2.71	0.0491
**PRSS33**	Homo sapiens protease, serine, 33 (PRSS33), mRNA [NM_152891]	2.70	0.0491
**RXRG**	Homo sapiens retinoid X receptor, gamma (RXRG), transcript variant 2, non-coding RNA [NR_033824]	2.69	0.0491
**lnc-ARHGEF40-1**	LNCipedia lincRNA (lnc-ARHGEF40-1), lincRNA [lnc-ARHGEF40-1:1]	2.67	0.0491

TABLE S2

List of Downregulated Genes in the cDNA Microarray Dataset

GeneSymbol	Description	Fold-change	*p*-value
**FOXL2**	Homo sapiens forkhead box L2 (FOXL2), mRNA [NM_023067]	-161.43	0.0456
**SPRR2D**	Homo sapiens small proline-rich protein 2D (SPRR2D), mRNA [NM_006945]	-122.24	0.0491
**STAT6**	Homo sapiens signal transducer and activator of transcription 6, interleukin-4 induced (STAT6), transcript variant 2, mRNA [NM_003153]	-102.51	0.0491
**BEST1**	Homo sapiens bestrophin 1 (BEST1), transcript variant 1, mRNA [NM_004183]	-99.86	0.0456
**TRPV6**	Homo sapiens transient receptor potential cation channel, subfamily V, member 6 (TRPV6), mRNA [NM_018646]	-95.98	0.0456
**SPRR2C**	Homo sapiens small proline-rich protein 2C (pseudogene) (SPRR2C), non-coding RNA [NR_003062]	-90.05	0.0491
**SPRR2E**	Homo sapiens small proline-rich protein 2E (SPRR2E), mRNA [NM_001024209]	-88.79	0.0491
**SLIT1**	Homo sapiens slit homolog 1 (Drosophila) (SLIT1), mRNA [NM_003061]	-82.74	0.0456
**lnc-RP11-597K23.2.1-2**	BC011509 Cox15 protein {Mus musculus} (exp=-1; wgp=0; cg=0), partial (5%) [THC2616036]	-81.40	0.0491
**TRPV6**	Homo sapiens transient receptor potential cation channel, subfamily V, member 6 (TRPV6), mRNA [NM_018646]	-79.76	0.0491
**GSE1**	Homo sapiens Gse1 coiled-coil protein (GSE1), transcript variant 1, mRNA [NM_014615]	-77.99	0.0456
**CREB5**	Homo sapiens cAMP responsive element binding protein 5 (CREB5), transcript variant 1, mRNA [NM_182898]	-64.42	0.0491
**LOC100507639**	Homo sapiens uncharacterized LOC100507639 (LOC100507639), long non-coding RNA [NR_121625]	-53.47	0.0456
**KCNG1**	Homo sapiens potassium channel, voltage gated modifier subfamily G, member 1 (KCNG1), mRNA [NM_002237]	-42.96	0.0456
**SPATA6L**	Homo sapiens spermatogenesis associated 6-like (SPATA6L), mRNA [NM_001039395]	-42.25	0.0456
**VIP**	Homo sapiens vasoactive intestinal peptide (VIP), transcript variant 1, mRNA [NM_003381]	-38.15	0.0491
**CCDC144NL-AS1**	PREDICTED: Homo sapiens uncharacterized LOC102724783 (LOC102724783), ncRNA [XR_424802]	-37.99	0.0456

**LOC101930053**	PREDICTED: Homo sapiens uncharacterized LOC101930053 (LOC101930053), transcript variant X1, ncRNA [XR_428440]	-33.87	0.0456
**IL20RB**	Homo sapiens interleukin 20 receptor beta (IL20RB), mRNA [NM_144717]	-33.04	0.0456
**LURAP1L**	Homo sapiens leucine rich adaptor protein 1-like (LURAP1L), mRNA [NM_203403]	-31.37	0.0456
**lnc-MAOA-2**	LNCipedia lincRNA (lnc-MAOA-2), lincRNA [lnc-MAOA-2:1]	-29.26	0.0491
**LOC101929174**	PREDICTED: Homo sapiens uncharacterized LOC101929174 (LOC101929174), ncRNA [XR_424270]	-25.96	0.0491
**SYPL2**	Homo sapiens synaptophysin-like 2 (SYPL2), mRNA [NM_001040709]	-24.39	0.0456
**TMIE**	Homo sapiens transmembrane inner ear (TMIE), mRNA [NM_147196]	-20.44	0.0456
**LINC237**	long intergenic non-protein coding RNA 237 [Source:HGNC Symbol;Acc:HGNC:38166] [ENST00000455890]	-20.20	0.0456
**QPCT**	Homo sapiens glutaminyl-peptide cyclotransferase (QPCT), mRNA [NM_012413]	-20.14	0.0491
**LOC101930048**	PREDICTED: Homo sapiens uncharacterized LOC101930048 (LOC101930048), transcript variant X1, ncRNA [XR_428438]	-19.80	0.0456
**FXYD4**	Homo sapiens FXYD domain containing ion transport regulator 4 (FXYD4), transcript variant 1, mRNA [NM_173160]	-19.40	0.0491
**LAMTOR5-AS1**	Homo sapiens LAMTOR5 antisense RNA 1 (LAMTOR5-AS1), long non-coding RNA [NR_102697]	-19.25	0.0456
**MOCOS**	Homo sapiens molybdenum cofactor sulfurase (MOCOS), mRNA [NM_017947]	-19.24	0.0456
**HCG26**	Homo sapiens HLA complex group 26 (non-protein coding) (HCG26), long non-coding RNA [NR_002812]	-19.02	0.0456
**ABCC8**	Homo sapiens ATP-binding cassette, sub-family C (CFTR/MRP), member 8 (ABCC8), transcript variant 2, mRNA [NM_000352]	-17.75	0.0492
**lnc-HSPB7-1**	LNCipedia lincRNA (lnc-HSPB7-1), lincRNA [lnc-HSPB7-1:1]	-16.55	0.0456
**KLHDC7B**	Homo sapiens kelch domain containing 7B (KLHDC7B), mRNA [NM_138433]	-16.26	0.0456
**CDH13**	Homo sapiens cadherin 13 (CDH13), transcript variant 1, mRNA [NM_001257]	-16.19	0.0456
**MOV10L1**	Homo sapiens Mov10 RISC complex RNA helicase like 1 (MOV10L1), transcript variant 1, mRNA [NM_018995]	-15.98	0.0456
**GDAP1L1**	Homo sapiens ganglioside induced differentiation associated protein 1-like 1 (GDAP1L1), transcript variant 2, mRNA [NM_024034]	-15.47	0.0491
**SARS**	seryl-tRNA synthetase [Source:HGNC Symbol;Acc:HGNC:10537] [ENST00000369923]	-15.44	0.0491
**PRSS3**	Homo sapiens protease, serine, 3 (PRSS3), transcript variant 2, mRNA [NM_002771]	-15.00	0.0456
**lnc-NDUFS6-8**	LNCipedia lincRNA (lnc-NDUFS6-8), lincRNA [lnc-NDUFS6-8:1]	-14.95	0.0491

**TAC4**	Homo sapiens tachykinin 4 (hemokinin) (TAC4), transcript variant alpha, mRNA [NM_170685]	-14.85	0.0491
**LOC100289026**	PREDICTED: Homo sapiens putative uncharacterized protein FLJ00310-like (LOC100289026), misc_RNA [XR_424060]	-14.34	0.0456
**BEX2**	Homo sapiens brain expressed X-linked 2 (BEX2), transcript variant 1, mRNA [NM_001168399]	-14.17	0.0491
**KIAA1257**	Homo sapiens KIAA1257 (KIAA1257), mRNA [NM_020741]	-14.04	0.0492
**ZAP70**	Homo sapiens zeta-chain (TCR) associated protein kinase 70kDa (ZAP70), transcript variant 1, mRNA [NM_001079]	-13.50	0.0491
**CPLX3**	Homo sapiens complexin 3 (CPLX3), mRNA [NM_001030005]	-13.19	0.0456
**ADAMTS14**	Homo sapiens ADAM metallopeptidase with thrombospondin type 1 motif, 14 (ADAMTS14), transcript variant 1, mRNA [NM_139155]	-13.08	0.0456
**PATL2**	Homo sapiens protein associated with topoisomerase II homolog 2 (yeast) (PATL2), mRNA [NM_001145112]	-12.90	0.0491
**LOC79999**	Homo sapiens uncharacterized LOC79999 (LOC79999), mRNA [NM_001291904]	-12.49	0.0456
**PLEKHG1**	Homo sapiens pleckstrin homology domain containing, family G (with RhoGef domain) member 1 (PLEKHG1), mRNA [NM_001029884]	-12.48	0.0456
**LOC101060442**	PREDICTED: Homo sapiens calcium-binding mitochondrial carrier protein SCaMC-1-like (LOC101060442), misc_RNA [XR_250827]	-12.44	0.0491
**IL2RG**	Homo sapiens interleukin 2 receptor, gamma (IL2RG), mRNA [NM_000206]	-12.38	0.0456
**LINC00632**	Homo sapiens long intergenic non-protein coding RNA 632 (LINC00632), transcript variant 3, long non-coding RNA [NR_104228]	-12.32	0.0491
**SLPI**	Homo sapiens secretory leukocyte peptidase inhibitor (SLPI), mRNA [NM_003064]	-12.01	0.0491
**KCNG1**	PREDICTED: Homo sapiens potassium voltage-gated channel, subfamily G, member 1 (KCNG1), transcript variant X2, mRNA [XM_006723786]	-11.63	0.0456
**SPINK1**	Homo sapiens serine peptidase inhibitor, Kazal type 1 (SPINK1), mRNA [NM_003122]	-11.60	0.0456
**SYNPO**	synaptopodin [Source:HGNC Symbol;Acc:HGNC:30672] [ENST00000394243]	-11.56	0.0456
**HPN**	Homo sapiens hepsin (HPN), transcript variant 1, mRNA [NM_182983]	-11.24	0.0491
**DST**	Homo sapiens dystonin (DST), transcript variant 1e, mRNA [NM_001723]	-11.11	0.0456
**CEACAM21**	Homo sapiens carcinoembryonic antigen-related cell adhesion molecule 21 (CEACAM21), transcript variant 3, mRNA [NM_001288773]	-11.09	0.0456
**TP53I11**	Homo sapiens tumor protein p53 inducible protein 11 (TP53I11), transcript variant 1, mRNA [NM_001258320]	-11.08	0.0456
**GLDC**	Homo sapiens glycine dehydrogenase (decarboxylating) (GLDC), mRNA [NM_000170]	-11.06	0.0456

**LOC400768**	PREDICTED: Homo sapiens uncharacterized LOC400768 (LOC400768), misc_RNA [XR_110483]	-11.01	0.0491
**MMP19**	Homo sapiens matrix metallopeptidase 19 (MMP19), transcript variant 1, mRNA [NM_002429]	-10.93	0.0456
**PLK5**	Homo sapiens polo-like kinase 5 (PLK5), mRNA [NM_001243079]	-10.89	0.0491
**LOC102724467**	Homo sapiens uncharacterized LOC102724467 (LOC102724467), long non-coding RNA [NR_120309]	-10.86	0.0491
**CHRND**	Homo sapiens cholinergic receptor, nicotinic, delta (muscle) (CHRND), transcript variant 1, mRNA [NM_000751]	-10.66	0.0491
**DST**	Homo sapiens dystonin (DST), transcript variant 1e, mRNA [NM_001723]	-10.62	0.0456
**ATXN7L1**	ataxin 7-like 1 [Source:HGNC Symbol;Acc:HGNC:22210] [ENST00000388807]	-10.15	0.0456
**LINC01300**	Homo sapiens long intergenic non-protein coding RNA 1300 (LINC01300), long non-coding RNA [NR_024441]	-10.01	0.0456
**PSMG3-AS1**	Homo sapiens PSMG3 antisense RNA 1 (head to head) (PSMG3-AS1), transcript variant 1, long non-coding RNA [NR_027329]	-9.97	0.0474
**DENND2D**	Homo sapiens DENN/MADD domain containing 2D (DENND2D), transcript variant 1, mRNA [NM_024901]	-9.93	0.0491
**CDX2**	Homo sapiens caudal type homeobox 2 (CDX2), mRNA [NM_001265]	-9.65	0.0456
**LINC00973**	long intergenic non-protein coding RNA 973 [Source:HGNC Symbol;Acc:HGNC:48868] [ENST00000473756]	-9.50	0.0491
**FRK**	Homo sapiens fyn-related Src family tyrosine kinase (FRK), mRNA [NM_002031]	-9.39	0.0491
**LOC100506737**	PREDICTED: Homo sapiens uncharacterized LOC100506737 (LOC100506737), ncRNA [XR_172388]	-9.37	0.0491
**AP3B2**	Homo sapiens adaptor-related protein complex 3, beta 2 subunit (AP3B2), transcript variant 2, mRNA [NM_004644]	-9.33	0.0456
**HAGLR**	Homo sapiens HOXD antisense growth-associated long non-coding RNA (HAGLR), transcript variant 1, long non-coding RNA [NR_110458]	-9.15	0.0456
**MAP1LC3B**	Homo sapiens microtubule-associated protein 1 light chain 3 beta (MAP1LC3B), mRNA [NM_022818]	-9.12	0.0456
**CSF1R**	Homo sapiens colony stimulating factor 1 receptor (CSF1R), transcript variant 1, mRNA [NM_005211]	-9.07	0.0491
**HRG**	Homo sapiens histidine-rich glycoprotein (HRG), mRNA [NM_000412]	-8.93	0.0491
**FAM122C**	Homo sapiens family with sequence similarity 122C (FAM122C), transcript variant 4, mRNA [NM_001170781]	-8.87	0.0491
**PELI2**	Homo sapiens pellino E3 ubiquitin protein ligase family member 2 (PELI2), mRNA [NM_021255]	-8.79	0.0459
**LTF**	Homo sapiens lactotransferrin (LTF), transcript variant 1, mRNA [NM_002343]	-8.36	0.0456
**CCDC144NL-AS1**	PREDICTED: Homo sapiens uncharacterized LOC102724783 (LOC102724783), ncRNA [XR_424802]	-8.27	0.0491
**NHS**	Homo sapiens Nance-Horan syndrome (congenital cataracts and dental anomalies) (NHS), transcript variant 1, mRNA [NM_198270]	-8.17	0.0491

**SLC22A15**	Homo sapiens solute carrier family 22, member 15 (SLC22A15), mRNA [NM_018420]	-8.07	0.0456
**LOC100499489**	Homo sapiens uncharacterized LOC100499489 (LOC100499489), long non-coding RNA [NR_036533]	-8.00	0.0491
**LOC100288798**	Homo sapiens uncharacterized LOC100288798 (LOC100288798), transcript variant 1, long non-coding RNA [NR_125377]	-7.97	0.0491
**LOC102724539**	Homo sapiens uncharacterized LOC102724539 (LOC102724539), long non-coding RNA [NR_125998]	-7.67	0.0456
**HHLA3**	Homo sapiens HERV-H LTR-associating 3 (HHLA3), transcript variant 3, mRNA [NM_001036646]	-7.57	0.0491
**PDCD1**	Homo sapiens programmed cell death 1 (PDCD1), mRNA [NM_005018]	-7.47	0.0491
**SLC44A5**	Homo sapiens solute carrier family 44, member 5 (SLC44A5), transcript variant 1, mRNA [NM_152697]	-7.46	0.0491
**CLCC1**	Homo sapiens chloride channel CLIC-like 1 (CLCC1), transcript variant 1, mRNA [NM_001048210]	-7.44	0.0456
**LETM2**	Homo sapiens leucine zipper-EF-hand containing transmembrane protein 2 (LETM2), transcript variant 2, mRNA [NM_144652]	-7.44	0.0491
**PARP12**	Homo sapiens poly (ADP-ribose) polymerase family, member 12 (PARP12), transcript variant 1, mRNA [NM_022750]	-7.40	0.0456
**DNAH1**	Homo sapiens dynein, axonemal, heavy chain 1 (DNAH1), mRNA [NM_015512]	-7.34	0.0456
**lnc-HPCAL1-1**	LNCipedia lincRNA (lnc-HPCAL1-1), lincRNA [lnc-HPCAL1-1:13]	-7.27	0.0456
**SIAE**	Homo sapiens sialic acid acetylesterase (SIAE), transcript variant 1, mRNA [NM_170601]	-7.26	0.0491
**CLCC1**	Homo sapiens chloride channel CLIC-like 1 (CLCC1), transcript variant 1, mRNA [NM_001048210]	-7.26	0.0456

Table S3

Minimum Energy Required for each LncRNA to Interact with the Predicted mRNAs as Calculated by the rtool Software

GNAS-AS1 (FC = 56.87)	
**mRNA**	**MinEnergy**
SYNPO	-41.8
GSE1	-30.8
ATXN7L1	-30.3
KCNG1	-29.6
SMOC2	-28.6
SPRR2E	-28.5
PRSS3	-26.9
HSPA1B	-25.9
CCDC106	-25.9
ABCC8	-25.9
STAT6	-25.7
ADAMTS14	-25.6
FHDC1	-25.5
CREB5	-24.3
CHRNE	-23.8
KLHDC7B	-23.7
SPRR2D	-23.6
FBP1	-23.3

LINC00668 (FC = 13.28)	
**mRNA**	**MinEnergy**
CRYBG3	-35.6
FAM212B	-34.0
ABCC8	-33.5
SYNPO	-33.2
RDX	-32.3
LIMA1	-31.6
SPATA6L	-31.1
SARS	-31.0
CRYBG3	-30.7
DST	-30.3
AP3B2	-29.5
KMT2B	-28.5
TRPV6	-28.5
BEX2	-28.1
RGS9	-27.9
LTF	-27.8
DNAH1	-27.0
C15ORF37	-26.1

MIR205HG (FC = 39.70)	
**mRNA**	**MinEnergy**
GDAP1L1	-37.1
PRG2	-36.8
DNAH1	-36.4
RGS9	-30.3
SARS	-30.1
HSPA1B	-29.9
DST	-29.8
SPRR2D	-29.0
SYNPO	-28.3
KCNG1	-28.0
CPLX3	-28.0
RDX	-27.8
FAM204A	-26.6
ADAMTS14	-26.5

PSMG3-AS1 (FC = -9.9)	
**mRNA**	**MinEnergy**
KLHDC7B	-34.2

LMCD1-AS1 (FC = -5.53)	
**mRNA**	**MinEnergy**
BEX2	-31.0
MMP19	-30.4
TMEM19	-29.8
CPLX3	-29.6
SYNPO	-29.2
FAM212B	-29.1
SARS	-28.3
KMT2B	-28.3
ABCC8	-26.5
DST	-26.1
RDX	-25.6
ZNF44	-25.5
FAM122C	-25.2
GLDC	-24.9
PLEKHG1	-24.8
MPP1	-24.6
DCAF8	-23.8
CREB5	-23.7
LTF	-23.0

PRG2	-32.2
PATL2	-31.4
TAP2	-30.4
KCNG1	-30.3
IL20RB	-30.0
MUC3A	-29.4
DST	-28.4
PDCD1	-27.9
EFNA3	-27.8
ZAP70	-27.1
PARP12	-26.9
FHDC1	-26.8

**LINC01006 (FC = -6.01)**	
**mRNA**	**MinEnergy**
ABCC8	-30.1
CSF1R	-28.6
CHRND	-28
DST	-27.3
ATXN7L1	-25.4
PER3	-25.0
PTPN22	-24.7
DNAH1	-24.6
ARRDC4	-24.5
CNTNAP3B	-23.8
ECEL1	-23.5
ADAMTS14	-23.3
AP3B2	-22.9
SPRR2E	-22.7
TAP2	-22.6
BEST1	-22.4
SLC22A15	-22.2
NOS3	-22.2
KCNG1	-22.0
KLHL29	-21.7
FHDC1	-21.4
OC90	-21.4

TUBB3	-22.9

`

**LINC00941 (FC = -6.21)**	
**mRNA**	**MinEnergy**
BEX2	-40.0
SARS	-33.8
IL20RB	-31.9
AP3B2	-30.1
ECEL1	-28.4
HRG	-28.2
FBP1	-27.5
MPP1	-27.1
DST	-26.2
CPLX3	-24.9
ZNF177	-24.6

**LINC00973 (FC = -9.50)**	
**mRNA**	**MinEnergy**
RTKN2	-31.5
HSPA1B	-28.1
DNAH1	-27.3
RGS9	-26.3
KIAA1257	-24.5
GDAP1L1	-23.7
VIP	-23.6
PDCD1	-23.2
OC90	-22.6
ZNF33B	-22.6
DENND2D	-22.0
MOCOS	-22.0
BEX2	-21.9
ST7L	-21.9
DST	-21.6
FAM122C	-21.2
CHRND	-21.2
ADAMTS14	-20.9
PTPN22	-20.7
MIOX	-20.7
STXBP3	-20.6
CLCC1	-20.5
LIMA1	-20.3
